# Inhaled mosliciguat (BAY 1237592): targeting pulmonary vasculature via activating apo-sGC

**DOI:** 10.1186/s12931-022-02189-1

**Published:** 2022-10-01

**Authors:** Eva M. Becker-Pelster, Michael G. Hahn, Martina Delbeck, Lisa Dietz, Jörg Hüser, Johannes Kopf, Thomas Kraemer, Tobias Marquardt, Thomas Mondritzki, Johannes Nagelschmitz, Sylvia M. Nikkho, Philippe V. Pires, Hanna Tinel, Gerrit Weimann, Frank Wunder, Peter Sandner, Joachim Schuhmacher, Johannes-Peter Stasch, Hubert K. F. Truebel

**Affiliations:** 1 Pharmaceuticals R&D, Pharma Research Center, Bayer AG, Aprather Weg 18A, 42113 Wuppertal, Germany; 2 Fakultät für Gesundheit, University Witten/Herdecke, Witten, Germany; 3 The Janssen Pharmaceutical Companies of Johnson & Johnson, Allschwil, Switzerland; 4 Department of Pharmacology, Hannover Medical School, Hannover, Germany; 5 Institute of Pharmacy, University Halle-Wittenberg, Halle, Germany; 6 The Knowledge House, Breite Strasse 22, Düsseldorf, Germany

**Keywords:** Mosliciguat, Nitric oxide-insensitive soluble guanylate cyclase, Pulmonary diseases, Soluble guanylate cyclase activator, Ventilation/perfusion mismatch

## Abstract

**Background:**

Oxidative stress associated with severe cardiopulmonary diseases leads to impairment in the nitric oxide/soluble guanylate cyclase signaling pathway, shifting native soluble guanylate cyclase toward heme-free apo-soluble guanylate cyclase. Here we describe a new inhaled soluble guanylate cyclase activator to target apo-soluble guanylate cyclase and outline its therapeutic potential.

**Methods:**

We aimed to generate a novel soluble guanylate cyclase activator, specifically designed for local inhaled application in the lung. We report the discovery and in vitro and in vivo characterization of the soluble guanylate cyclase activator mosliciguat (BAY 1237592).

**Results:**

Mosliciguat specifically activates apo-soluble guanylate cyclase leading to improved cardiopulmonary circulation. Lung-selective effects, e.g., reduced pulmonary artery pressure without reduced systemic artery pressure, were seen after inhaled but not after intravenous administration in a thromboxane-induced pulmonary hypertension minipig model. These effects were observed over a broad dose range with a long duration of action and were further enhanced under experimental oxidative stress conditions. In a unilateral broncho-occlusion minipig model, inhaled mosliciguat decreased pulmonary arterial pressure without ventilation/perfusion mismatch. With respect to airway resistance, mosliciguat showed additional beneficial bronchodilatory effects in an acetylcholine-induced rat model.

**Conclusion:**

Inhaled mosliciguat may overcome treatment limitations in patients with pulmonary hypertension by improving pulmonary circulation and airway resistance without systemic exposure or ventilation/perfusion mismatch. Mosliciguat has the potential to become a new therapeutic paradigm, exhibiting a unique mode of action and route of application, and is currently under clinical development in phase Ib for pulmonary hypertension.

**Supplementary Information:**

The online version contains supplementary material available at 10.1186/s12931-022-02189-1.

## Introduction

Impaired nitric oxide (NO) and cyclic guanosine monophosphate (cGMP) signaling have been implicated in the pathogenesis of cardiopulmonary diseases [1]. NO stimulates the heme-containing soluble guanylate cyclase (sGC), triggering cGMP production resulting in pulmonary smooth muscle relaxation. Furthermore, anti-inflammatory, anti-fibrotic, and anti-proliferative effects have been reported with sGC activators [2]. Therefore, pharmacologic modulation of the NO–sGC–cGMP pathway was one of the first therapeutic strategies in pulmonary hypertension (PH) with phosphodiesterase type 5 inhibitors (PDE5is) (sildenafil and tadalafil), which prevent degradation of cGMP, approved as treatment for patients with pulmonary arterial hypertension (PAH) [3–6]. More recently, a new class of drugs, sGC stimulators, which directly stimulate the native sGC enzyme and stabilize NO binding to sGC has been developed. The sGC stimulator riociguat is approved as treatment for PAH and for chronic thromboembolic pulmonary hypertension (CTEPH) that is inoperable by pulmonary endarterectomy (PEA) or with persistent/recurrent PH after PEA [6–10], and vericiguat demonstrated positive results in a phase III heart failure trial and was very recently approved for patients with symptomatic chronic heart failure [11, 12]. Furthermore, inhaled NO (iNO) is used to treat a spectrum of cardiopulmonary conditions, including PH in children and adults. However, its widespread use is not only limited by its short half-life but also by logistical and financial barriers, and NO resistance in up to 40% of patients [13–19], likely because of an altered sGC expression or shift to apo-sGC [20]. In addition, the duration of pulmonary vasodilation by iNO is very short, NO is rapidly scavenged by oxyhemoglobin in red blood cells [21], and there is frequent rebound pulmonary vasoconstriction after iNO is discontinued. At high concentrations, non-specific interactions with various biomolecules are reported [22–24].

**Fig. 1:**
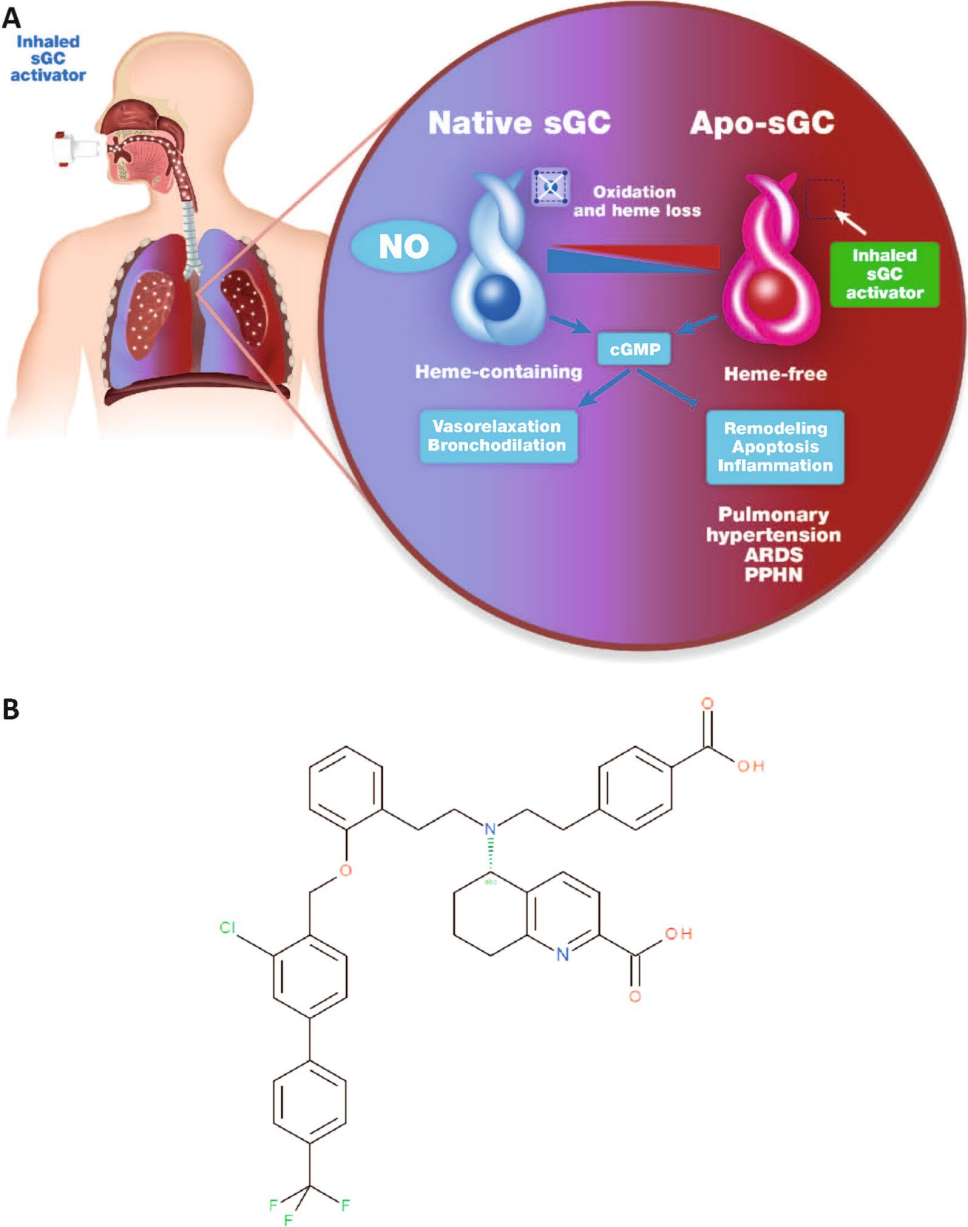
**A** NO and sGC activators target two different redox states of sGC: the NO-sensitive reduced native sGC and the NO-insensitive oxidized and finally heme-free apo-sGC, respectively. NO stabilizes the nitrosyl-heme complex of the reduced sGC to increase cGMP. Activators of sGC such as mosliciguat (**B**) bind to the unoccupied heme-binding complex or displace the prosthetic heme of sGC. The inhaled sGC activator is activating the heme-free apo-sGC to increase cGMP which mediates vasorelaxation and bronchodilation and blocks remodelling, apoptosis, and inflammation. *ARDS* acute respiratory distress syndrome, *cGMP* cyclic guanosine monophosphate, *NO* nitric oxide, *PPHN* persistent pulmonary hypertension in the neonate, *sGC* soluble guanylate cyclase

There is growing evidence that an sGC redox equilibrium exists and that oxidative stress associated with many cardiopulmonary diseases shifts intracellular levels of native sGC towards the heme-free apo-sGC form, which is unresponsive to NO, making vasodilator therapy with NO and PDE5is less effective (Fig. 1A) [2]. Even sGC stimulators, independent of NO, require heme-containing sGC and are less effective at targeting apo-sGC [25]. The discovery of sGC activators binding and activating apo-sGC was a milestone in NO–sGC–cGMP pharmacology and is still a matter of intensive research [26, 27].

Here we report the discovery and pharmacologic characterization of a novel sGC activator, mosliciguat (BAY 1237592), specifically tailored for inhaled application in patients with PH.

## Methods

Mosliciguat (BAY 1237592) (5-{[2-(4-carboxyphenyl)ethyl][2-(2-{[3-chloro-4‘(trifluoro-methyl)biphenyl-4-yl]methoxy}phenyl)ethyl]amino}-5,6,7,8-tetrahydroquinoline-2-carboxylic acid) (Fig. 1B) was synthesized as described in patent WO2014012934 [28].

### sGC assay

sGC was purified using a baculovirus/Sf9 expression system and enzyme activity measured in the presence of Mg^2+^ as described previously [29].

Animal studies are reported in compliance with the Animal Research: Reporting of in Vivo Experiments (ARRIVE) v2.0 guidelines [30] and with the recommendations made by the *British Journal of Pharmacology* [31].

### PH minipig model

Ellegaard Göttinger minipigs (bred in Dalmose, Denmark; female, with 4–5 kg body weight) were sedated, anesthetized, and assessed as described previously [32]. Intubated animals were artificially ventilated (room air enriched with 40% oxygen; constant volume [VT] of 10–12 mL/kg [Avea ventilator, Viasys Healthcare, Conshohocken, PA, USA, or Engström Carestation, GE Healthcare, Solingen, Germany]; 35 breaths/min), to maintain an end-tidal carbon dioxide concentration of approximately 4% (Capnomac^®^ Ultima carbon dioxide monitor, Nova Med GmbH, Lonnerstadt, Germany). To induce PH, a thromboxane A2 analog infusion (15–45 µg/kg/h U-46619) was used [33, 34] to elevate mean pulmonary arterial pressure (PAP) above 35 mmHg. Mosliciguat was given by inhalation using a nebulizer (Aeroneb^®^Pro Nebulizer, Inspiration Medical GmbH, Bochum, Germany) connected to the inspiration side of the ventilation system at 7 min/dose. Mosliciguat was dissolved in vehicle solution (0.2% citric acid; pH 9 [volume 0.75 mL/kg in the nebulization unit]). For infusions, mosliciguat was dissolved in an infusion vehicle composed of dimethyl sulphoxide:transcutol:polyethylene glycol 400:NaCl solution (1:35:35:29% [v/v]). For combination experiments, bosentan (1 mg/kg bolus [0.5 mL/kg] followed by an infusion of 1 mg/kg/h [0.5 mL/kg/h]) or sildenafil infusion (300 µg/kg/h, 0.5 mL/kg/h) both dissolved in infusion vehicle was started and 1 h later mosliciguat (30 µg/kg) was inhaled. Similarly, 1H-[1,2,4]Oxadiazolo[4,3-a]quinoxalin-1-one (ODQ; 1 mg/kg bolus [0.5 mL/kg]) or N(G)-Nitro-l-arginine methyl ester (L-NAME) (10 mg/kg bolus followed by a continuous infusion of 5 mg/kg/h) was dissolved in an infusion vehicle [35, 36]. Cardiovascular parameters were collected using the PoNeMah acquisition and analysis system (Data Sciences International, St. Paul, MN, USA) through Gould transducers (series 6600). Mean values were sampled during stable intervals of 1–3 min.

### Hypoxic dog model

To investigate the hemodynamic response of inhaled mosliciguat during experimental PH, we used a conscious dog model of hypoxia-induced PH [37]. Briefly, three male beagle dogs (Marshall BioResources, USA) (8–15 kg) were assessed with telemetry sensors (Data Science International, St Paul, MN, USA) to measure PAP non-invasively. (Gender mixture makes group housing of dogs difficult in terms of animal welfare.) After wound healing, animals were trained for nebulization and the hypoxia procedure. On experimental days 1, 5, 12, 16, and 24 h after drug administration (either inhaled mosliciguat 100 µg/kg or vehicle solution), dogs were placed into a sling and measurements were performed as previously described [37]. For the vehicle control at 24 h, the 1-h vehicle control from the prior day was re-used to minimize stress on the dogs.

### Univentilated lung model

Seven-week-old Ellegaard Göttinger minipigs (Dalmose, Denmark; female, 4–5 kg body weight) were sedated, anesthetized, and assessed, and cardiovascular parameters were collected as previously described [32]. (Experiments in minipigs were performed in females because non-invasive catheterization of the bladder for conductance of anesthesia is impossible for male pigs. In addition, group housing in the animal facility is not possible with a mix of genders.) Right-sided unilateral ventilation was induced by advancing the tracheal tube into the right main bronchus followed by inflation of the cuff balloon. Tube placement into the right main bronchus was confirmed by auscultation. Ventilation was adjusted for unilateral ventilation by increasing the breath frequency to 40 breaths/min and by calculating the tidal volume using the following formula: VT (during unilateral ventilation) = VT (during bilateral ventilation) × breath frequency (during bilateral ventilation)/breath frequency (during unilateral ventilation).

In each animal, two cycles of 10 min of unilateral ventilation were followed by 30 min of bilateral ventilation without pharmacologic intervention to verify reproducibility of unilateral ventilation (Additional file 1: Fig. S1). Following these control cycles, each animal underwent six repetitive cycles of unilateral ventilation (10 min) starting with two vehicle reference cycles (one vehicle inhaled, and another vehicle given as intravenous bolus injection) followed by 30 min of regular bilateral ventilation, respectively. After these cycles, four intervention cycles were performed. Animals were separated into two groups of three or four animals in which vehicle and mosliciguat (100 µg/kg inhaled nominal dose and 30 µg/kg and 100 µg/kg intravenously) were compared in the following intervention cycles (Additional file 1: Fig. S1). In the mosliciguat-treated group, inhaled mosliciguat was administered in the unilateral ventilation cycle 5; inhalation took about 7 min. Unilateral ventilation cycle 6 was performed 90 min after the start of mosliciguat inhalation to allow the drug to reach its maximal effect. Cumulative intravenous bolus administration of mosliciguat was then applied 15 min before unilateral ventilation cycles 7 and 8, respectively, to ensure stable hemodynamic conditions and adequate drug distribution. In the vehicle-treated group, the appropriate vehicle solutions were applied at the equivalent time points.

Hemodynamic parameters (e.g., mean PAP, blood pressure, heart rate, and oxygen saturation [SaO_2_]) were monitored continuously. Effects on oxygenation parameters (e.g., area under the SaO_2_ curve) as well as hemodynamic parameters were compared with vehicle-treated animals. Methods for mosliciguat inhaled and intravenous application are identical to those used in the PH minipig model.

### Bronchoconstriction model

The bronchoconstriction model [38] used male Brown Norway rats (Charles River, Sulzfeld, Germany; 10–12 weeks old) randomized to three treatment groups (mosliciguat 1 µg/kg, 10 µg/kg, and 100 µg/kg; *n* = 12–13), one positive control group (tiotropium 1 µg/kg; *n* = 12), and one vehicle control group (*n* = 17). Animals were treated with the inhaled test compounds 60 min before provocation. Mosliciguat, vehicle, or tiotropium were aerosolized using a micro feeding system and a dispersion nozzle operated with pressurized air. The aerosol concentration was determined by filter sampling with gravimetric analysis and monitored continuously by a photometer. The lung-deposited doses were assessed from the inhalation dose [39]. The generation of acetylcholine aerosols for the provocation tests was performed as previously described (Bronchy Type III and a Fraunhofer ITEM dispersion nozzle) [40–42]. After treatment and an additional pause to meet the 60-min time-period from the end of treatment to the beginning of acetylcholine provocation, animals were anaesthetized (intraperitoneal 80 mg/kg ketamine and 4 mg/kg xylazine) for orotracheal intubation and placed in a body plethysmograph. Oxygen was adjusted to approximately 40%. After reaching a steady state in respiration, lung function of the spontaneously breathing animal was recorded as baseline values for ≥ 2 min; among other parameters, lung resistance and dynamic compliance were used to assess bronchoconstriction. Data recording and processing were performed using HEM software, version 4.2 (Notocord Systems, Croissy, France). After recording baseline values 60 ± 15 min after inhalational treatment, the animals were provoked with acetylcholine (5% aqueous solution aerosol). The assessment of lung function was continued during and for ≥ 3 min after the exposure.

### Data analysis and statistics

The data and statistical analyses comply with the recommendations on experimental design and analysis in pharmacology. It was technically not possible to perform preclinical experiments in pigs and dogs in a randomized and blinded manner because the study team performing the experiment was also applying the drug. According to our long experience in performing underlying experimental settings and in accordance with animal protection, groups were composed of three to four animals to provide physiologically meaningful results. Statistics were only performed with preliminary characteristics due to the low number of animals.

#### PH minipig model

Mean values of cardiovascular parameters were sampled during stable intervals of 1–3 min. Differences between mosliciguat and vehicle were analyzed using the paired *t*-test performed with Microsoft Excel, comparing mean values at different time points between vehicle- and mosliciguat-treated animals.

#### Hypoxic dog model

To evaluate the duration of effect of mosliciguat, decreases in mean PAP during the hypoxia challenge were calculated for all animals at different time points as: (SUM of mean PAP from 10 to 40 min of mosliciguat-treated animal) – (SUM of mean PAP from 10 to 40 min of respective vehicle-treated animal)/number of time points.

#### Bronchoconstriction model

Bronchodilatory efficacy of the test compounds was compared with the vehicle control group and the positive control tiotropium group by means of parametric tests. For two groups, Student’s *t*-test was used; for multiple comparisons (> 2 groups),  analysis of variance (ANOVA) and parametric Dunnett test were performed. To assess the efficacy, the inhibition of acetylcholine-induced bronchoconstrictive responses was calculated. For the absolute maximum or minimum values of the lung function parameters, the inhibition was calculated as the difference between the respective treatment group and the positive control group in percent of the response range of the positive control (which is the delta value between baseline and the maximum or minimum of the parameter). For example, if the mean value of maximum lung resistance (RL) of a treatment group reached the baseline value of the control group, the inhibition would be 100%, and if it was equal to the maximum RL value of the control group the inhibition would be 0%.

## Results

### Discovery of mosliciguat

A targeted medicinal chemistry effort led to the discovery of a novel class of potent heme- and NO-independent sGC activators with mosliciguat (Fig. 1B) as the lead clinical development candidate [32, 35, 36]. By extensive structure–activity relationship exploration of the structural class of bicarboxylic acids [36], we attempted to improve physicochemical and pharmacokinetic properties in order to enhance lung selectivity over a broad dose range. Through these efforts, we discovered mosliciguat, which could be administered directly via inhalation.

### In vitro sGC characterization

**Fig. 2:**
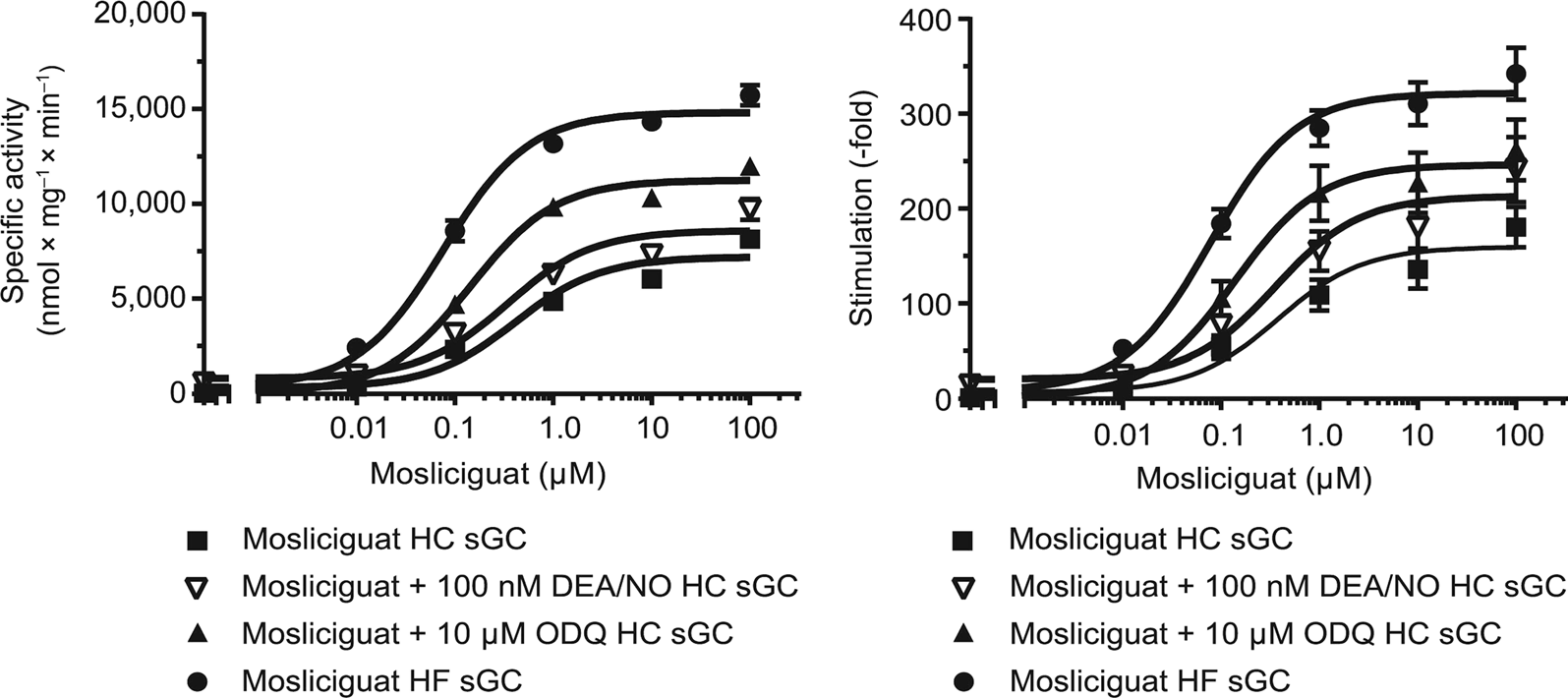
The effect of mosliciguat with DEA/NO and ODQ on isolated HC and HF rat sGC in vitro specific activity (left) and stimulation (right). *DEA/NO* diethylammonium (Z)-1-(*N*,*N*-diethylamino)diazen-1-ium-1,2-diolate, *HC* heme-containing, *HF* heme-free, *ODQ* 1H-[1,2,4]Oxadiazolo[4,3-a]quinoxalin-1-one,* sGC* soluble guanylate cyclase

The results of the in vitro characterization of sGC are shown in Fig. 2. Mosliciguat activated recombinant sGC in a concentration-dependent manner (0.01–100 µM) with an effect of 8.3- to 180.5-fold. The NO-releasing drug, diethylammonium (Z)-1-(*N*,*N*-diethylamino)diazen-1-ium-1,2-diolate (DEA/NO) at a concentration of 0.1 µM induced a maximal increase in sGC activity of 14.4-fold. In combination, mosliciguat (0.01–100 µM) and DEA/NO (0.1 µM) showed an additive effect over a wide range of concentrations. In the presence of the sGC inhibitor ODQ, an increase in sGC activity up to 261.7-fold was observed with mosliciguat. In contrast to NO donors, mosliciguat still activated heme-free sGC concentration-dependently from 0.01 to 100 µM with an effect of 52.5- to 342.0-fold. Thus, mosliciguat is able to activate apo-sGC independently of NO.

### Anaesthetized PH minipig model

**Fig. 3:**
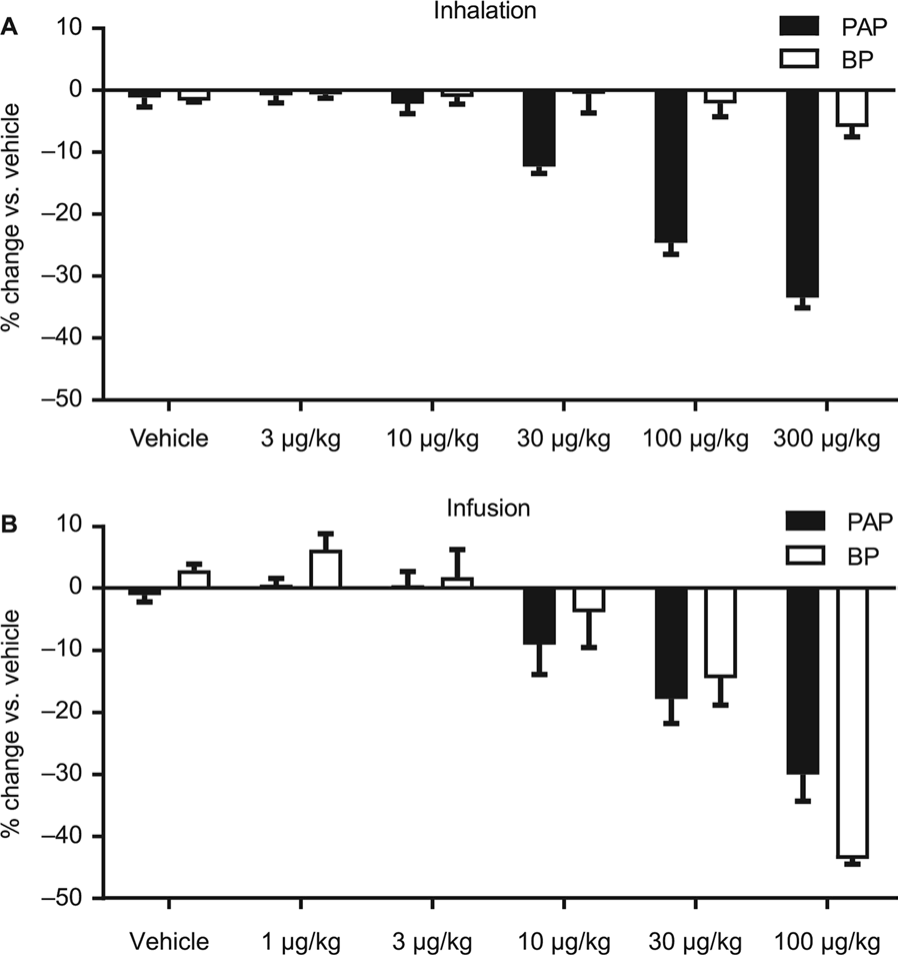
**A** Percentage changes in PAP (mean) and BP (mean) versus vehicle-treated animals after inhaled cumulative application of mosliciguat under thromboxane A2-induced PH in minipigs observed 30 min after inhalation of cumulative doses. Data are mean ± SEM (*n* = 3). **B** Percentage changes on PAP (mean) and BP (mean) versus vehicle-treated animals after cumulative infusions of mosliciguat under thromboxane A2-induced PH in minipigs observed 30 min after infusion of cumulative doses. Data are mean ± SEM (*n* = 3). *BP* mean blood pressure, *PH* pulmonary hypertension, *PAP* mean pulmonary arterial pressure, *SEM* standard error of the mean

Mosliciguat was intensively characterized in various small and large animal models. To evaluate its efficacy on the pulmonary and systemic circulation, but also its feasibility as an inhaled drug, an anesthetized minipig PH model (thromboxane A2 challenge) was used. The effects of mosliciguat on mean blood pressure (BP) and mean PAP after inhaled application of 3, 10, 30, 100, and 300 µg/kg cumulative doses were compared with the effects of mosliciguat after intravenous infusion of 1, 3, 10, 30, and 100 µg/kg cumulative doses. After inhalation, mosliciguat induced a dose-dependent decrease in PAP starting at the nominal dose of 10 µg/kg (Fig. 3A). No relevant effects were observed on BP (Fig. 3A). After short-term intravenous infusions, a concomitant dose-dependent decrease in BP and PAP, starting at 10 µg/kg, was observed (Fig. 3B). Although inhaled mosliciguat 300 µg/kg did not result in a relevant reduction of BP, 30 µg/kg given intravenously decreased both BP and PAP. These data indicate the high selectivity of inhaled mosliciguat for PAP reduction and thereby lung selectivity.

**Fig. 4:**
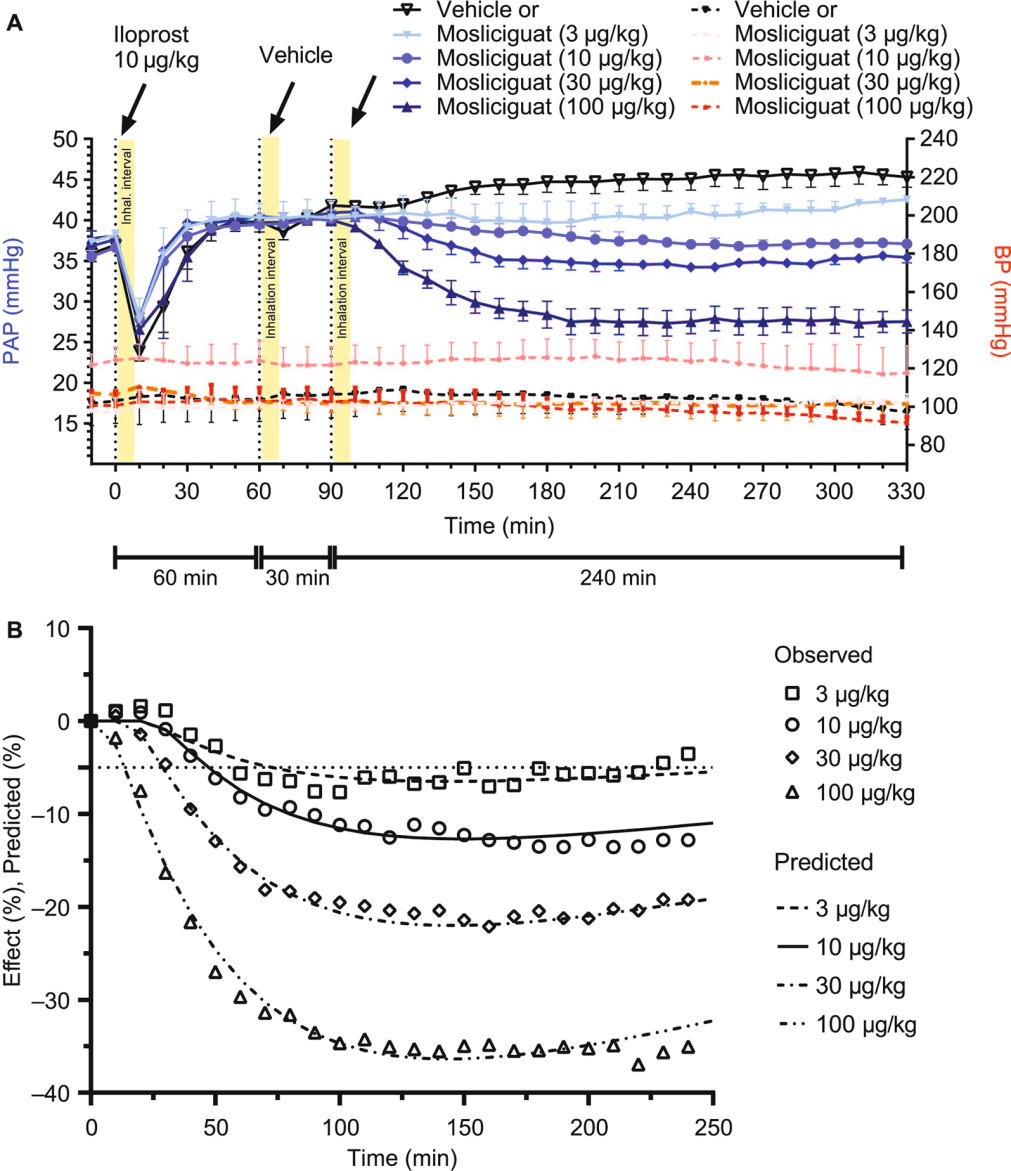
**A** Effects of mosliciguat after inhaled application under thromboxane A2-induced PH in minipigs compared with iloprost inhalation as clinical reference. **B** Observed (symbols) and predicted (lines) PAP (mean) values after administration of 0.15, 0.5, 1.5, and 5 µg/kg mosliciguat (lung-deposited doses; figure shows inhalation doses) to minipigs (7-min inhalation as liquid aerosol). Data are mean ± SEM (*n* = 3–4). PAP (mean) reduction of 5% indicated as straight dotted line. *BP* mean blood pressure, *PH* pulmonary hypertension, *PAP* mean pulmonary arterial pressure, *SEM* standard error of the mean

The decrease in PAP showed a slow onset, reaching maximum decrease 90–120 min after inhalation. This was maintained over the 4-h observation period without any effects on BP (Fig. 4A). Compared with inhaled iloprost, the maximal PAP effect was comparable or even greater with mosliciguat, but the duration was more than eight times longer. Of the nominal effective inhaled mosliciguat doses (3–100 µg/kg), 3 µg/kg was considered to be the minimal effective dose resulting in a > 5% PAP reduction compared with vehicle-treated animals, which is considered a relevant effect regarding physiological changes in PAP (Fig. 4B) [43].

**Fig. 5:**
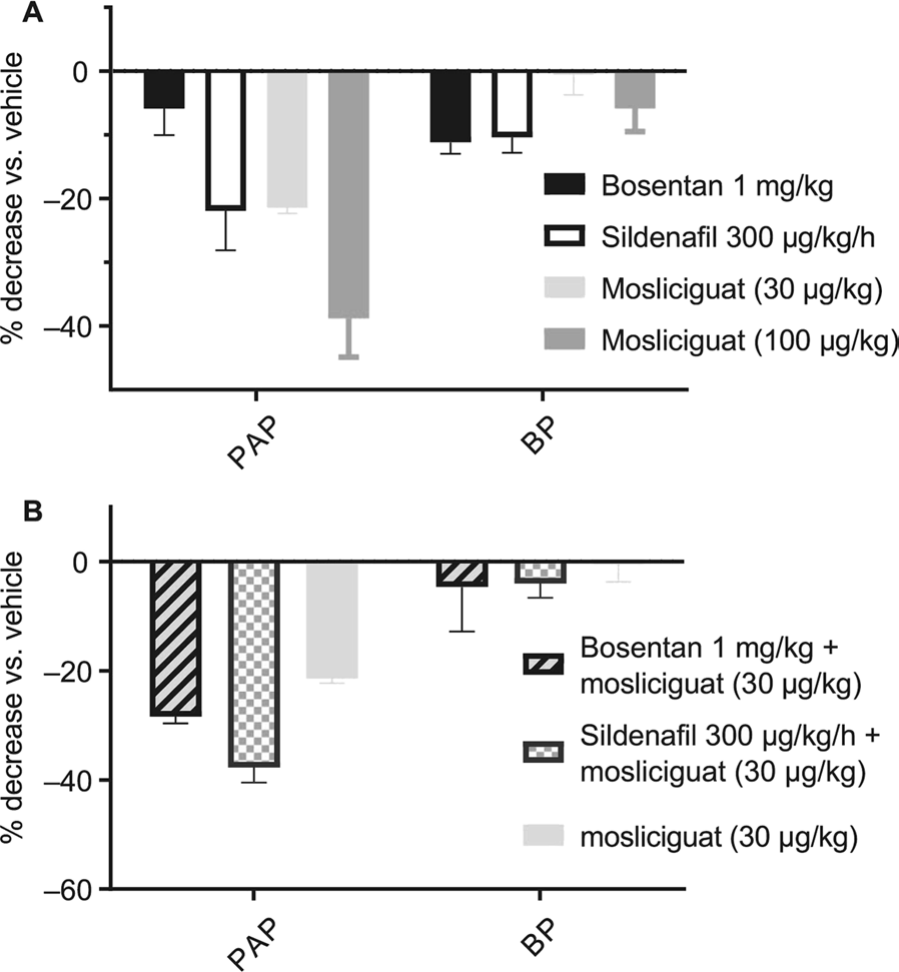
**A** Efficacy of inhaled mosliciguat on PAP (mean) and BP (mean) compared with efficacy of systemic applied bosentan and sildenafil at steady-state concentrations. **B** Efficacy of inhaled mosliciguat on PAP (mean) and BP (mean) in combination with bosentan or sildenafil under constant infusions. Results show percentage changes versus vehicle-treated control animals as mean ± SEM out of three or four experiments. *BP* mean blood pressure, *PAP* mean pulmonary arterial pressure, *SEM* standard error of the mean

In addition, efficacy of inhaled mosliciguat was compared with sildenafil and bosentan (Fig. 5A). Bosentan led to a decrease in BP with only minor effects on PAP, whereas infusion of sildenafil led to decreases in both PAP and BP. Inhaled mosliciguat led to similar (30 µg/kg) or even larger (100 µg/kg) effects on PAP with no or minor effects on BP. Importantly, mosliciguat (30 µg/kg) maintained its efficacy regarding PAP when combined with either sildenafil or bosentan compared with mosliciguat treatment alone without additional effects on BP (Fig. 5B).

**Fig. 6:**
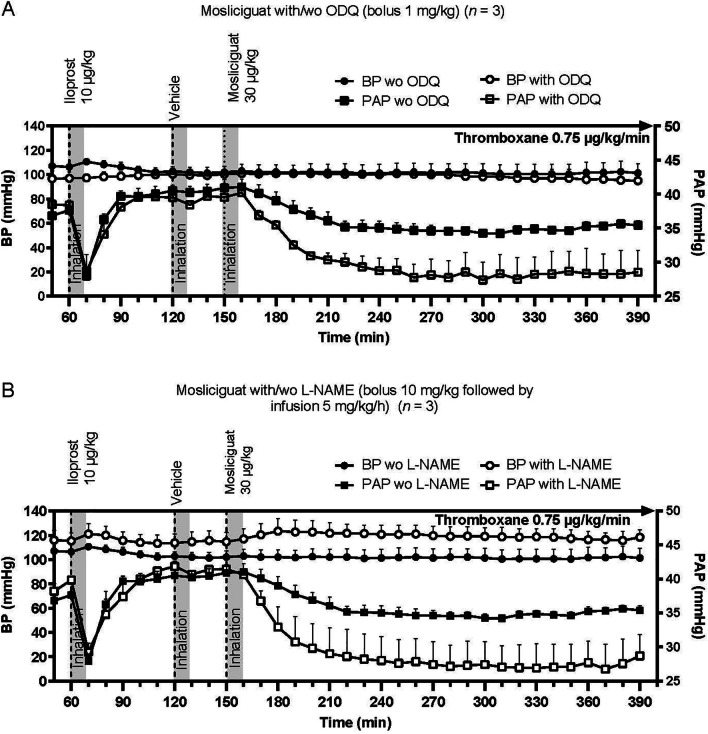
**A** Effects of inhaled mosliciguat under thromboxane A2-induced PH in minipigs with and without pretreatment by ODQ. **B** Effects of mosliciguat after inhaled application under thromboxane A2-induced PH in minipigs with and without pretreatment by L-NAME. Data are mean ± SEM (*n* = 3). *BP* mean blood pressure, *L-NAME* N(G)-Nitro-l-arginine methyl ester, *ODQ* 1H-[1,2,4]Oxadiazolo[4,3-a]quinoxalin-1-one, *PAP* mean pulmonary arterial pressure, *SEM* standard error of the mean, *wo* without

Under oxidative stress conditions (i.e., low NO) induced by pretreatment with either ODQ or L-NAME, the effects of inhaled mosliciguat (30 µg/kg) on PAP were enhanced without any effects on BP (Fig. 6A and B). Such an enhancement was not observed with iloprost.

### Conscious hypoxic dogs

**Fig. 7:**
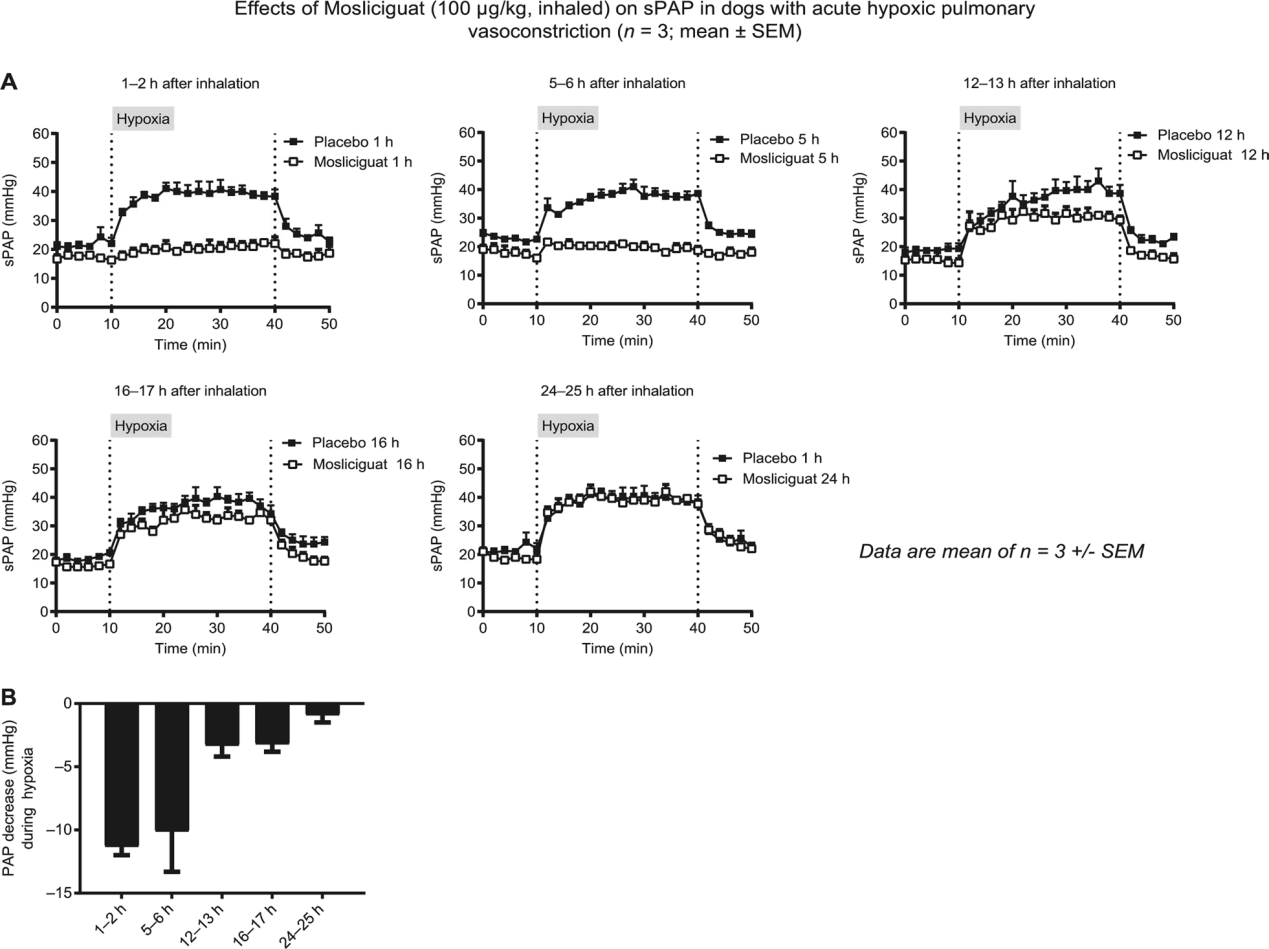
**A** Effects of mosliciguat after inhaled application on systolic PAP during hypoxia-induced vasoconstriction in conscious dogs (*n* = 3). **B** Mean PAP decrease evoked by mosliciguat after inhaled application during hypoxia challenge in conscious dogs. PAP decrease for each animal is calculated as follows: PAP decrease = (SUM of mPAP from 10–40 min of mosliciguat-treated animal) ‒ (SUM of mPAP from 10–40 min of respective vehicle-treated animal)/number of time points. *mPAP* mean pulmonary arterial pressure, *PAP* mean pulmonary arterial pressure, *SEM* standard error of the mean, *sPAP* systolic pulmonary arterial pressure

We evaluated the lung-selective and duration of effects of mosliciguat under conscious conditions, in another species, and under a more physiologic stimulus for PAP increase. Dogs were exposed to hypoxia for 30 min to induce a hypoxia-mediated systolic PAP increase, and mosliciguat (100 µg/kg) was applied by inhalation 1, 5, 12, 16, and 24 h before hypoxia. Compared with vehicle-treated animals, attenuation of the hypoxia-induced systolic PAP increase was observed for 1–17 h with mosliciguat and dissipated after 24 h (Fig. 7A and B). No effects on heart rate were observed (data not shown).

### Univentilated lung model for the evaluation of arterial oxygenation as a proxy for ventilation/perfusion mismatch

**Fig. 8:**
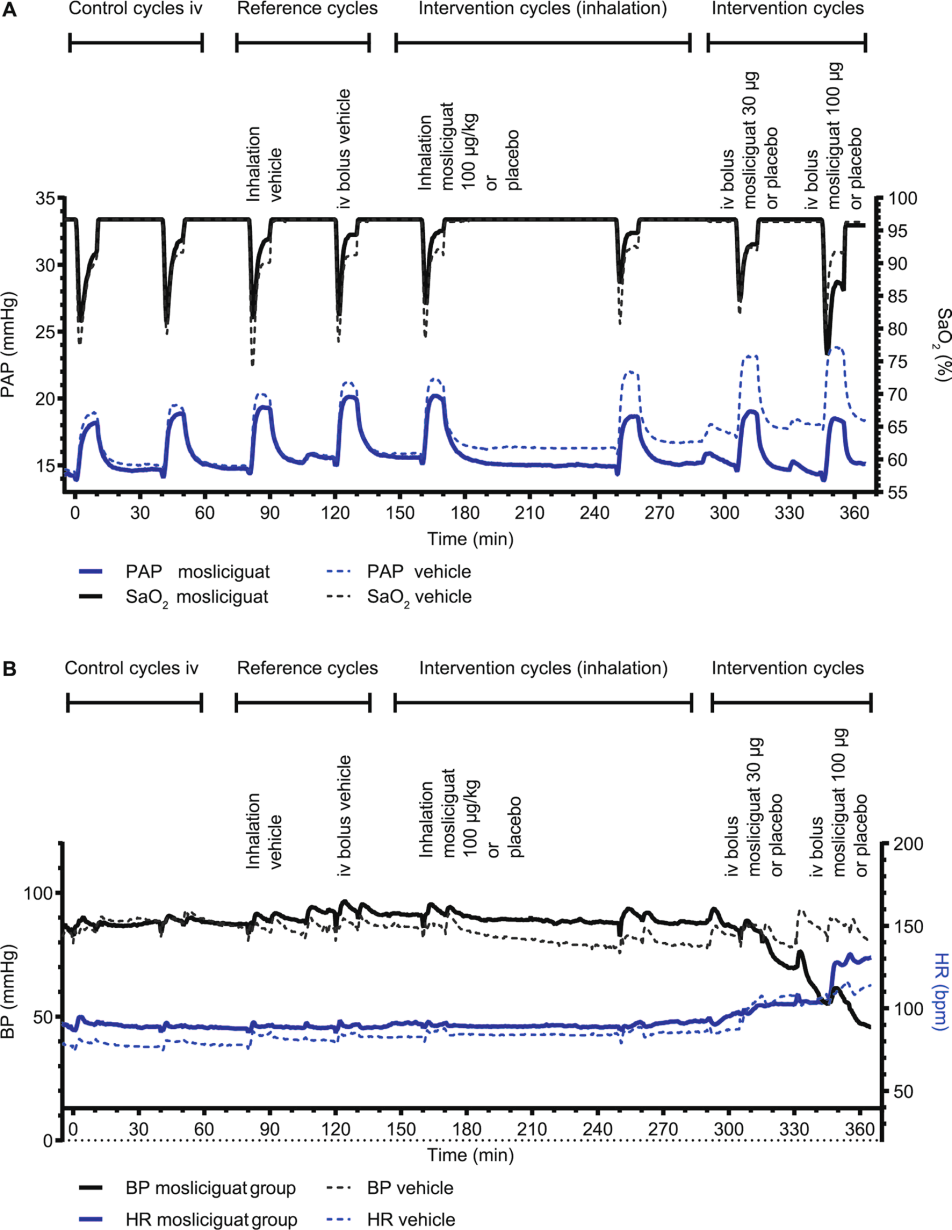
**A** Effects of mosliciguat after inhaled and intravenous application and vehicle administration on PAP (mean) and SaO_2_ during eight broncho-occlusion cycles in minipigs. **B** Effects of mosliciguat after inhaled and intravenous application and vehicle administration on BP (mean) and HR during eight broncho-occlusion cycles in minipigs. Data are mean (*n* = 3 or 4). *BP* mean blood pressure, *HR* heart rate, *PAP* mean pulmonary arterial pressure, *SaO*_*2*_ arterial oxygen saturation of hemoglobin

In the minipig model we measured the intrapulmonary selectivity of mosliciguat. In vehicle-treated anesthetzsed minipigs, repetitive 10-min cycles of unilateral lung ventilation resulted in a reproducible increase in PAP (mean), accompanied by decreases in SaO_2_ (Fig. 8A). Heart rate and BP were stable during the study period with inhaled mosliciguat (Fig. 8B), whereas intravenous mosliciguat (30 and 100 µg/kg) induced a BP decrease. In contrast to vehicle-treated animals, animals treated with inhaled mosliciguat showed a trend toward smaller desaturation areas (intervention cycles [inhalation]) as well as a decrease in PAP under normoxic and broncho-occlusion cycles, which was even more pronounced 90 min after inhalation (intervention cycle 2 [inhalation]) (Fig. 8A). Intravenous mosliciguat led to an increase in desaturation area as well as a further reduction in PAP compared with vehicle-treated animals (intervention cycles [intravenous]) (Fig. 8A).

**Fig. 9:**
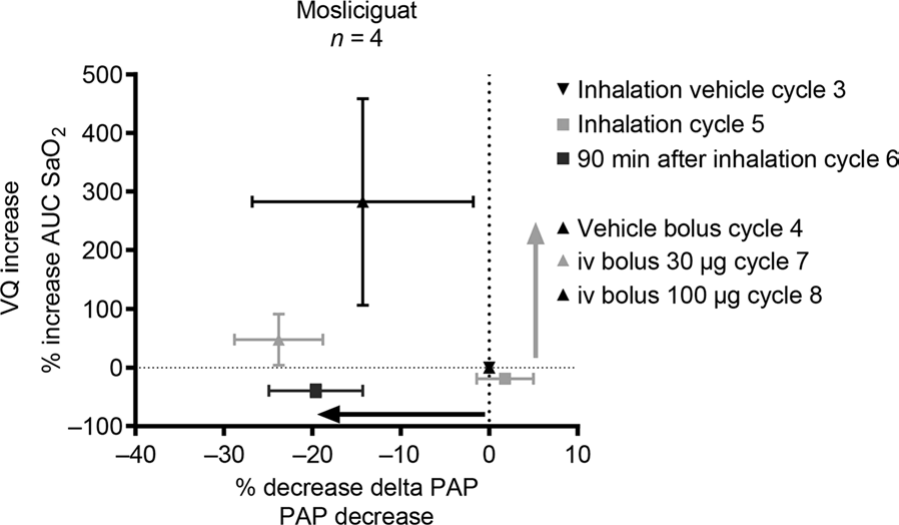
Mosliciguat capacity to decrease maximal hypoxic PAP (mean) (positive treatment effect) and AUC SaO_2_ (unwanted desaturation effect) based on effects of representative cycles (*n* = 4 animals); data are mean ± SEM (*n* = 4). Mosliciguat inhaled (100 µg/kg nominal dose); intravenous 30 and 100 µg/kg. *AUC SaO*_*2*_ area under the SaO_2_ curve, *PAP* mean pulmonary arterial pressure, *SaO*_*2*_ arterial oxygen saturation of hemoglobin, *SEM* standard error of the mean, *VQ* ventilation/perfusion

Percentage changes in maximal hypoxic PAP (with reduction as the desired effect) and unwanted desaturation (area under the SaO_2_ curve) were compared to determine the risk–benefit ratio of inhaled versus intravenous application of mosliciguat (Fig. 9). As reference cycles, cycle 3 for the inhaled and cycle 4 for the intravenous application were used. During unilateral ventilation cycles, hypoxia-induced increases in PAP were reduced by mosliciguat after inhaled (cycle 6) as well as after intravenous application (cycles 7 and 8). No reduction was observed in cycle 5, due to the simultaneous application. Inhaled mosliciguat did not increase desaturation area, but rather showed a trend towards a reduction of the desaturation area.

In summary, inhaled mosliciguat effectively reduced PAP in the unilateral broncho-occlusion model. In contrast to systemic administration, no negative effect on desaturation area could be detected after inhalation, providing a better risk–benefit ratio for this route of administration.

### Bronchoconstriction rat model

**Fig. 10:**
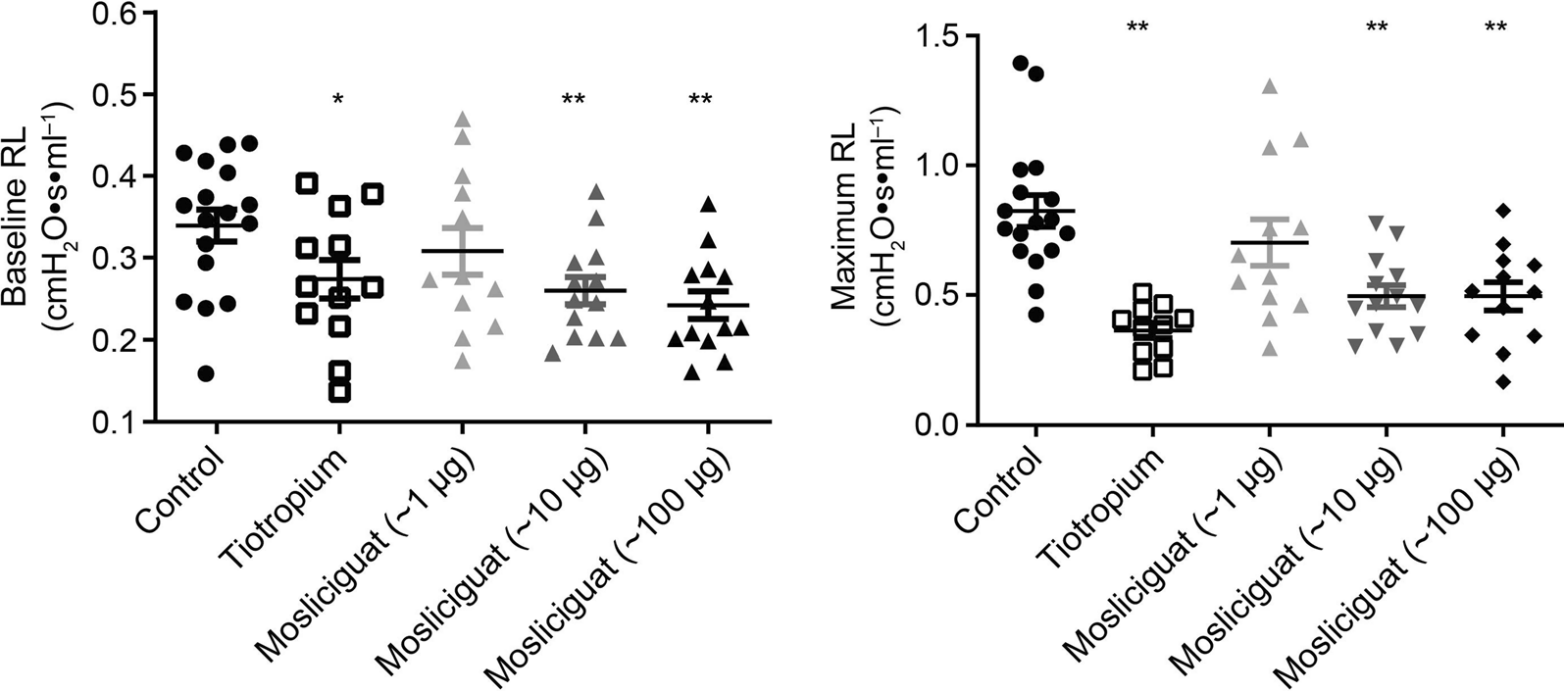
Left: bronchodilating effects on lung resistance at baseline prior to acetylcholine provocation (mean ± SEM, *p < 0.05, **p < 0.01 versus positive control group). Right: bronchoprotective effects after acetylcholine-induced bronchospasm: maximum lung resistance during and after provocation. Values are given as individual data and mean ± SEM with *n* = 11–17 animals/group and **p < 0.01 versus positive control group. *RL* lung resistance, *SEM* standard error of the mean

Lung function was measured in anesthetized rats at baseline and after bronchoconstriction (acetylcholine provocation). To quantify the bronchoconstrictive response to acetylcholine, increases in RL and decreases in dynamic compliance were evaluated. The long-acting cholinergic M3 receptor blocker tiotropium was used as positive control, and inhibited acetylcholine-induced bronchospasm, with RL decreased (Fig. 10). Mosliciguat also induced a dose-dependent (1, 10, and 100 µg/kg) bronchodilating effect reaching 68% for RL and 37% for dynamic compliance at the highest dose, suggesting an effect on the large as well as the small airways.

## Discussion

This is the first report of the discovery and pharmacologic properties of the sGC activator mosliciguat specifically designed for local application in the lung to treat PH. Inhaled mosliciguat specifically activates apo-sGC, leading to lung-selective effects, such as reduced PAP without reduced systemic artery pressure, over a broad dose range with a long duration of action. In contrast to systemic treatments, decreased PAP is achieved without ventilation/perfusion mismatch. With respect to airway resistance, mosliciguat shows additional beneficial bronchodilatory effects. Therefore, inhaled mosliciguat may overcome treatment limitations in patients with PH by improving pulmonary circulation and airway resistance without systemic exposure or ventilation/perfusion mismatch.

The efficacy of most standard-of-care PAH treatments is limited by systemic side effects, drug–drug interactions, and lack of disease modification, while combination therapy is often necessary and common clinical practice [6, 44–46]. A high unmet medical need therefore remains for treating the different forms of PH. Prostacyclins (e.g., iloprost, treprostinil) are the most commonly used inhaled agents to treat PAH; however, they require frequent administration (four to nine times daily) [6], which can reduce compliance. Inhaled mosliciguat was designed to overcome these treatment gaps.

Two distinct compound classes capable of modulating sGC were discovered at Bayer: sGC stimulators and sGC activators [1, 29, 36]. Both classes directly bind to sGC as allosteric modulators. sGC stimulators have a dual mode of action, directly stimulating native sGC independently of NO and also sensitizing sGC to low levels of NO by stabilizing NO–sGC binding. In contrast, sGC activators bind to the unoccupied heme-binding domain, thereby mimicking NO-bound heme, and activate the pathologically changed, NO-unresponsive apo-sGC (Fig. 1). Recent evidence has shown that oxidative stress associated with many cardiopulmonary diseases shifts intracellular native sGC levels towards the apo-sGC form [47, 48], providing the rationale for sGC activators [49–52] in cardiopulmonary diseases and PH.

In fact, in the persistent pulmonary hypertension in the neonate (PPHN) animal model, lower basal cGMP levels in pulmonary arterial smooth muscle cells were observed despite increased sGC α_1_- and β_1_-subunit protein expression [17]. Under these oxidative stress conditions, NO-induced cGMP generation was markedly reduced, whereas the effects of the sGC activator cinaciguat were increased. Moreover, cinaciguat-induced pulmonary vasodilation was significantly increased in fetal lambs after ductus arteriosus ligation [17]. After birth, cinaciguat caused a significantly greater fall in pulmonary vascular resistance than 100% oxygen, iNO, or acetylcholine, underlining the hypothesis of increased apo-sGC in the pathogenesis of PPHN [17]. These data suggest a significant therapeutic effect of sGC activators under pathophysiologic conditions of PH, acute respiratory distress syndrome (ARDS), and maybe even ARDS due to COVID-19 [53].

Therefore, a targeted intense medicinal chemistry effort was undertaken to identify an sGC activator for local application in the lung to treat diverse forms of PH, leading to the discovery of mosliciguat. Microparticles composed of cinaciguat have been reported to produce dose-dependent selective pulmonary vasodilation [36]. Additional inhaled sGC activators were therefore explored. Beginning with bicarboxylic acids, over 240 compounds were synthesized to improve the physicochemical, pharmacologic, and pharmacokinetic properties of the resulting lead compound, mosliciguat, with the aim of enhancing lung selectivity over a broad dose range without losing potency and avoiding special formulation requisites for use via commercially available nebulizers and/or dry powder inhalers.

Mosliciguat showed the typical sGC activator profile as observed with cinaciguat [54] and after inhaled application led to the desired highly selective dose-dependent decrease in PAP without concomitant effects on systemic BP. A physiologically relevant overspill of the drug was only observed at the highest dose, leading to a non-significant decrease in systemic BP. In the PH minipig model, the reduction of PAP with inhaled mosliciguat was comparable to that with inhaled iloprost but with a much (more than eightfold) longer duration of action. Of note, the duration of treatment effect with iloprost in our animal model correlated well with the duration of iloprost efficacy in clinical practice [55]. If this translation also holds true for the inhaled sGC activator, it may be the first inhalative drug in PH, with a twice- or once-daily regimen.

The efficacy of inhaled mosliciguat was compared with bosentan and sildenafil after systemic application in the PH minipig model. While mosliciguat resulted in a pulmonary-specific treatment effect (i.e., PAP reduction) without effects on BP, bosentan led to a decrease in BP with only minor effects on PAP, and sildenafil decreased both PAP and BP. Thus, the ratio of PAP to BP decreases for inhaled mosliciguat is superior to both PAH therapies in our animal model.

The pulmo-selective effects of inhaled mosliciguat were also observed in conscious dogs under a more physiologic hypoxia challenge already used in the development of PDE5is [56]. Therefore, in both species, a significant, selective, and long-lasting effect on pulmonary circulation was observed with mosliciguat. Interestingly, an inhaled sGC stimulator (MK-5475) also showed significant lung selectivity in a preclinical rat PH model [57] and is under clinical development in PAH (INSIGNIA-PAH (NCT04732221)): this supports the concept of extraordinary lung selectivity of inhaled sGC agonists. However, MK-5475 is an sGC stimulator, targeting the native sGC. This may have limitations in cardiopulmonary diseases under persisting oxidative stress. iNO, which also targets native sGC, loses its efficacy under oxidative stress; this probably causes high no-responder rates, e.g., in PPHN or ARDS [13–19]. It will be very interesting to see the clinical implications of the different modes of action between mosliciguat and MK-5475 in severe cardiopulmonary diseases.

When mosliciguat was combined with bosentan or sildenafil in the PH minipig model, the efficacy of mosliciguat was maintained without any unwanted effects on BP or kinetic interactions. Furthermore, under oxidative stress or low NO conditions, iloprost induced a short selective reduction in PAP without effects on BP, whereas the selective PAP effects of mosliciguat were enhanced.

Importantly, in contrast to sGC stimulators losing their efficacy under oxidative stress, the sGC activator demonstrated maintained or even enhanced efficacy in animal models. This supports the concept that mosliciguat could be a highly innovative cardiopulmonary therapy which might be superior to current PH therapies under disease conditions. However, this needs to be verified under clinical conditions such as PH, PPHN, or ARDS to confirm if responder rates are significantly higher for mosliciguat compared with iNO.

sGC activation focused on ventilated areas after inhaled application may selectively and potently decrease vasoconstriction in the lungs without influence on ventilation/perfusion mismatch. In the unilateral broncho-occlusion model, inhaled mosliciguat decreased PAP to a similar or even greater extent than intravenous mosliciguat without any unwanted effects on the observed desaturation areas; indeed, a trend towards a decrease in desaturation areas was observed with inhaled mosliciguat. These observations may be due to the slow tissue penetration of mosliciguat (data not shown).

Previous studies have shown that orally administered PAH drugs (e.g., bosentan, sildenafil, riociguat) dose-dependently decreased hypoxic PAP (i.e., a positive treatment effect) but increased the desaturation area (i.e., an unwanted desaturation effect) [32]. In contrast, inhaled mosliciguat showed similar or even higher efficacy regarding PAP reduction with no deterioration in the desaturation area. As a result, mosliciguat showed strong pulmonary and intrapulmonary selectivity after inhaled application. All these characteristics are of paramount importance in patients with ARDS and PPHN. For example, iNO shows a high non-responder rate in these conditions; this might be related to the increased oxidative stress, which shifts the equilibrium of the native sGC which mediates iNO effects towards the NO-insensitive apo-sGC. Therefore, we are convinced that a drug such as mosliciguat, targeting the apo-sGC and providing pulmonary and intrapulmonary selectivity, might be of advantage in these indications. However, this might require a different formulation providing more rapid adjustment to individual patient needs in the intensive care unit (ICU) than can be achieved with a dry powder inhaler. Moreover, our data indicate that mosliciguat not only improved circulation but also showed bronchodilatory properties which may be beneficial in the treatment of PH with chronic lung diseases or have potential for the treatment of asthma [58–60].

Therefore, mosliciguat might be a suitable inhaled drug which targets ventilated areas of the lung and evokes vasodilation only in ventilated areas, thereby overcoming this limitation of current PAH treatment options, for ARDS, or possibly COVID-19 patients who suffer from exaggerated ventilation/perfusion mismatch [53].

## Conclusion

Mosliciguat directly targets the NO-insensitive form of sGC in the lung and is the first sGC activator specifically designed for inhalation. Mosliciguat exhibits a unique mode of action and route of application resulting in high pulmonary efficacy and intrapulmonary selectivity. By improving pulmonary vascular as well as airway resistance without systemic side effects or any negative influence on ventilation/perfusion mismatch and showing an additional bronchodilatory effect, mosliciguat may overcome limitations of current PH therapies with the potential to become an efficient treatment option even in different forms of PH such as group III (PH due to lung diseases and/or hypoxia) which is currently not treatable. Mosliciguat is currently under clinical development in phase Ib as an inhaled therapy for PH.

## Supplementary Information

**Additional file 1: Fig. S1.** Treatment scheme of inhaled and systemic administered mosliciguat and of vehicle treatment in the minipig model. Each animal underwent eight unilateral ventilation cycles and animals were divided into two groups.

## Data Availability

The data that support the findings of this study are available from the corresponding author upon reasonable request. Some data may not be made available because of privacy or ethical restrictions.

## Abbreviations

ARDS: Acute respiratory distress syndrome
AUC SaO_2_: Area under the SaO_2_ curve
BP: Blood pressure
cGMP: Cyclic guanosine monophosphate
DEA/NO: Diethylammonium (Z)-1-(*N*,*N*-diethylamino)diazen-1-ium-1,2-diolate
HC: Heme-containing
HF: Heme-free
HR: Heart rate
iNO: Inhaled NO
l-NAME: N(G)-Nitro-l-arginine methyl ester
mPAP: Mean pulmonary arterial pressure
NO: Nitric oxide
ODQ: 1H-[1,2,4]Oxadiazolo[4,3-a]quinoxalin-1-one
PAH: Pulmonary arterial hypertension
PAP: Pulmonary arterial pressure
PDE5i: Phosphodiesterase type 5 inhibitors
PH: Pulmonary hypertension
PPHN: Persistent pulmonary hypertension in the neonate
RL: Lung resistance
SaO_2_: Arterial oxygen saturation of hemoglobin
SEM: Standard error of the mean
sGC: Soluble guanylate cyclase
sPAP: Systolic pulmonary arterial pressure
VQ: Ventilation/perfusion
