# Supplementary material for: Inhaled mosliciguat (BAY 1237592): targeting pulmonary vasculature via activating apo-sGC
Source: Respir Res. 2022 Oct 1;23:272. doi: 10.1186/s12931-022-02189-1 (PMC9526466; doi:10.1186/s12931-022-02189-1)
Supplement: Supplementary file 1 — Additional file 1: Fig. S1. Treatment scheme of inhaled and systemic administered mosliciguat and of vehicle treatment in the minipig model. Each animal underwent eight unilateral ventilation cycles and animals were divided into two groups. [file 12931_2022_2189_MOESM1_ESM.pdf]

Online Data Supplement

Inhaled mosliciguat (BAY 1237592): Targeting pulmonary vasculature via Apo-soluble guanylate cyclase

Eva M. Becker-Pelster | Michael G. Hahn | Martina Delbeck | Lisa Dietz | Jörg Hüser | Johannes Kopf | Thomas Kraemer | Tobias Marquardt | Thomas Mondritzki | Johannes Nagelschmitz | Sylvia M. Nikkho | Philippe V. Pires | Hanna Tinel | Gerrit Weimann | Frank Wunder | Peter Sandner | Joachim Schuhmacher | Johannes-Peter Stasch | Hubert K.F. Truebel

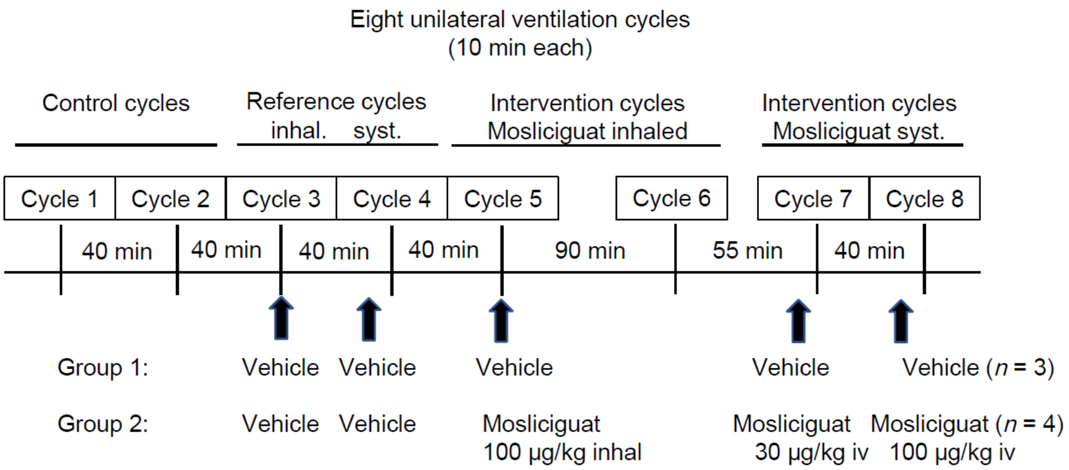

Fig. S1 Treatment scheme of inhaled and systemic administered mosliciguat and of vehicle treatment in the minipig model. Each animal underwent eight unilateral ventilation cycles and animals were divided into two groups.
